# Using theory of change frameworks to develop evaluation strategies for research engagement: results of a pre‐pilot study

**DOI:** 10.1002/jia2.25181

**Published:** 2018-10-18

**Authors:** Kathleen M MacQueen, Natalie T Eley, Mike Frick, Carol Hamilton

**Affiliations:** ^1^ Global Health Research FHI 360 Durham NC USA; ^2^ Treatment Action Group New York NY USA

**Keywords:** good participatory practice, community engagement, stakeholder engagement, evaluation, theory of change, clinical trials

## Abstract

**Introduction:**

Inadequate community and stakeholder engagement can lead to accusations that research is unethical and can delay or slow research or translation of results to practice. Such experiences have led major funders as well as regulatory and advisory bodies to establish minimal requirements for community and stakeholder engagement in HIV and other clinical research. However, systematic efforts to formally evaluate the contributions and impact of particular practices are lacking.

**Methods:**

A theory of change framework aligned with Good Participatory Practice for TB clinical trials was used to develop a set of measures for use in a minimally burdensome survey of trial implementing sites. The survey was pre‐piloted with three TB trial sites in North America, South America and Asia to assess the feasibility of surveying global research sites in a systematic way, and to see if the measures captured informative variation in the use of engagement strategies and desired outcomes. Surveys were conducted at baseline and six months. In‐depth interviews were conducted with site staff prior to the baseline survey to understand how sites conceptualized the concepts underlying the framework and the extent to which they viewed their work as aligned with the framework.

**Results:**

Survey measures captured considerable variability in the intensity and variety of engagement strategies, both across sites and within sites over time, and moderate variability in outcomes. Interviews indicated that underlying concepts were often unfamiliar to staff at baseline, but the goals of engagement aligned well with existing values.

**Conclusions:**

Brief, targeted surveys of trial sites to characterize use of broad strategies, specific practices and some outcomes are a feasible option for evaluating good participatory practice. Additional testing is warranted to assess and enhance validity, reliability and predictive value of indicators. Options for collecting outcome measures through additional objective means should be explored.

## 1 Introduction

Phenomenal progress has been made in the prevention and treatment of HIV and its comorbidities due in large part to the willingness of hundreds of thousands of people worldwide to volunteer as research participants. This is not to say that engaging participants has been easy. For HIV research, inadequate engagement has led to accusations of unethical behaviour and delayed or slowed research and translation of results to practice 1. Such controversies reflect historically based concerns about the potential for exploitation of vulnerable populations and persons due to the pervasive social, economic and political realities that travel with the HIV pandemic 2. These challenges, and their solutions, are not exclusive to HIV 3, 4, 5, 6. In the case of TB, an important comorbidity of HIV, there is the risk of similar emergent ethical controversies related, for example, to concerns about drug trials that fail to result in the roll‐out of successful products because the drugs are prohibitively expensive 7. The special challenges faced in implementing paediatric multidrug‐resistant TB clinical trials have also been noted 8. These experiences have led major funders as well as regulatory and advisory bodies to establish minimal requirements for community and stakeholder engagement in clinical research on the presumption that such engagement will bolster ethical practice and reduce the risk of trial disruption 9. While various community engagement strategies have been used in clinical trials, there has been little formal evaluation of their contribution to achieving ethical and scientific goals beyond case studies and exploratory assessments 9, 10, 11, 12, 13, 14, 15, 16, 17, 18, 19, 20.

A model increasingly used for implementing engagement in HIV, TB and other infectious disease clinical trials is the Good Participatory Practice (GPP) model 21, 22, 23, 24. GPP was first developed in 2007 as part of a broader response to controversial biomedical HIV prevention trials and then revised in 2011 22, 25. In October 2012, the Stakeholder and Community Engagement Workgroup (SCE‐WG) of the Critical Path to TB Drug Regimens (CPTR) issued Good Participatory Practice Guidelines for TB Drug Trials (GPP‐TB) 21, 26. This provided a unique opportunity to develop an evaluation framework for community engagement strategies for achieving ethical goals in a clinical trial context where such strategies were not already established practice. We undertook this objective by using a theory of change (TOC) approach to develop a framework for evaluating GPP‐TB 27, 28. TOC approaches emphasize techniques that are collaborative, participatory, and practical or applied; as such, TOC was well aligned with the explicit values of the Good Participatory Practice model. In contrast with a more general process evaluation approach for community participation 29, TOC frameworks link practices to outcomes and explicitly hypothesize why particular practices are expected to generate specific outcomes. The practices advocated for in GPP models are derived largely from anecdotal evidence, experiential learning and value statements. TOC provided a means for placing this rich history, discussion and consensus into a framework aligned with evaluation standards. Other examples of the use of TOC to develop evaluation strategies are comprehensively described by Breuer and colleagues, who also provide a checklist for reporting use of TOC in public health interventions 30. A major challenge faced in evaluating GPP is the lack of dedicated funding for this purpose, which means that the work is incremental and not fully aligned with the ideal scenario set out in the Breuer et al. checklist.

We developed a GPP TOC after the release of the GPP‐TB guidance, rather than as part of the GPP‐TB development process, a factor that others have noted as presenting evaluation challenges 31. Mitigating this challenge is the fact that development of GPP training programmes is also an ongoing, iterative process. Our efforts to develop a GPP TOC framework have been undertaken with these broader efforts to build GPP capacity globally.

**Figure 1:**
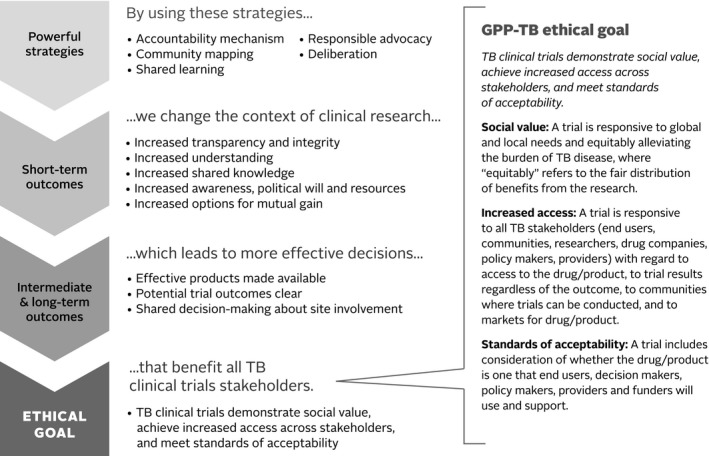
Theory of change framework for evaluating good participatory practice for TB clinical trials. A set of powerful strategies, each comprised of a range of potential practices, are hypothesized to lead to short‐term, intermediate and long‐term outcomes that cumulatively result in achieving the elements outlined in the Good Participatory Practice‐TB ethical goal. To qualify as powerful, a convincing argument or causal hypothesis had to be made for how a proposed strategy would lead to outcomes that in turn would lead to achieving the ethical goal 32.

In alignment with the TOC approach, we firstly sought consensus in defining a clear ethical goal of GPP‐TB, secondly worked backwards to identify appropriate and reasonable participatory strategies (noted as powerful strategies in the model) hypothesized to achieve the goal and thirdly used an iterative process to refine the framework. We established a project advisory board and brought together board members with other global TB clinical trials stakeholders for a two‐day meeting in Decatur, GA, USA in October 2013. The timing and location were chosen to take advantage of the annual meeting of the Community Research Advisors Group (CRAG) of the Centers for Disease Control and Prevention (CDC)‐sponsored TB Trials Consortium (TBTC). Following the meeting, the evaluation framework was refined through ongoing discussion with members of the project advisory board. The full model is briefly outlined in Figure 1; a comprehensive description of the framework is provided elsewhere 32.

With input from the project advisory board, we developed a set of measures mapped to the five participatory strategies and selected outcomes that could be used in a minimally burdensome survey of trial staff at implementing sites. Framework development included iterative discussion of how practices reflective of the powerful strategies could generate outcomes that would cumulatively lead to achieving the ethical goal. In developing the strategy measures we hypothesized that use of a greater variety of practices associated with a powerful strategy may be necessary to increase the effectiveness of the strategy for achieving the GPP‐TB goal. We further hypothesized that some practices may be necessary for achieving the GPP‐TB goal, independent of the intensity of practices. For example, use of a greater variety of engagement practices may be necessary for some outcomes, while the simple fact of having an established community advisory board or similar mechanism may be sufficient for achieving other outcomes.

Moving from the conceptual exercise of developing the TOC framework to developing appropriate evaluation measures, we explored feasibility of using a low‐burden survey as a core data collection mechanism. A priority in the survey design was to generate descriptive empirical data on the strategies and practices in use as well as the absence of use. No such systematic data currently exist. We also wanted to assess the feasibility of incorporating simple outcome indicators in this kind of survey. The work presented here is therefore an incremental step towards a comprehensive measurement approach, which would require the use of mixed methods (e.g. surveys, ethnographic observation, document review) and data from multiple sources beyond clinical trial research sites (e.g. community organizations, local gatekeepers and leaders, trial participants, trial sponsors and funders). In this pre‐pilot we were not able to test any hypotheses; this work represents an exploratory first step, including lessons learned and recommendations for implementing systematic multisite evaluations of engagement processes and outcomes.

## 2 Methods

The research was reviewed and approved by FHI 360's Protection of Human Subjects Committee and by ethics review committees at the South America and Asia sites; the North American site's IRB deemed the research exempt. The unit of analysis for this study was the research site. We did not collect identifying information about clients or patients, clinical trial research participants, individual research staff or individual stakeholders. Data collection consisted exclusively of information describing community and stakeholder engagement activities and practices undertaken and research outcomes experienced by each research site. We obtained oral informed consent at the time of the qualitative interviews (an accepted strategy for minimal risk studies that do not collect identifiable information on participants). For the survey, the informed consent language was provided in the email invitation and again at the beginning of the online survey, with responding to the online survey considered consent to participate. All data collection focused on obtaining information about the research sites’ practices and outcomes; no individual‐level data were solicited. Individual incentives for participation were not provided, rather, participating sites were provided funding to cover staff support for the various research activities. Data collection took place between May 2015 and April 2016.

### 2.1 Measurement

Survey indicators and measures are fully described in Data S1, including reference to the TOC framework components, the rationale for each indicator measure, the range of values associated with indicators, the wording of questions for each measure, how values were calculated from responses and the response items within measures.

#### 2.1.1 Strategy measures

The measures of the powerful strategies focused on a combination of binary yes/no indicator items and summary scores of the number of specific practices used by a site. The summary scores provided measures of the intensity of practice for a given strategy. One ranked item measure was included for the Deliberation Strategy, to assess the extent to which effort was made to include broad stakeholder perspectives in decision‐making. Table 1 provides an overview of the indicator, summary and ranked item measures for each of the five powerful strategies as well as definitions for each strategy; a more detailed breakdown is provided in Data S1.

**Table 1:** Baseline (BL) and follow‐up (FU) responses to survey to assess use of participatory strategies and associated practices at three sites

Participatory strategy & brief definition	Indicators	Examples of practices included in scores	Site A	Site B	Site C
***Accountability mechanisms*** Ensure transparency and ownership of the research process so that stakeholders achieve outcomes of integrity and efficacy through shared information	Is there a community advisory board (CAB) or similar mechanism? (Y/N)		BL = Y FU = N	BL = N FU = N	BL = Y FU = Y
	Summary score for CAB‐specific practices (range 6 to 60)	Outreach mechanisms used to recruit members; diversity of stakeholder membership	BL = 19 FU = n/a	BL = n/a FU = n/a	BL = 25 FU = 18
	Summary score for general engagement practices (range 0‐43)	Diversity of outreach mechanisms; updates provided in preferred language	BL = 5 FU = 0	BL = 14 FU = 14	BL = 11 FU = 8
***Community mapping*** Establishes a description of the local context (ethnographic mapping), identifies needs (cyclical) and develops an understanding of community to ensure research is mutually beneficial. Also describes the research context and the global public health context as they relate to TB, to understand the opportunities, needs and constraints within which research agendas are developed, funded and implemented	Summary score for community mapping (range 0 to 38)	Staff can readily identify local leaders where participants reside and track global debates relevant to TB	BL = 15 FU = 12	BL = 16 FU = 15	BL = 17 FU = 16
***Shared learning*** Provides awareness raising among all stakeholders & encompasses communication and engagement strategies. Measures of success may include mitigation of misconceptions about research, community contributions to research protocols and the language/vocabulary used to describe studies, enhanced stakeholder ownership of trials and/or the research process, transparency and accountability/efficiency/complementarity	Summary score for shared learning (range 0 to 51)	Community stakeholders participate in research team meetings; information from conferences shared with stakeholders	BL = 36 FU = 32	BL = 22 FU = 31	BL = 34 FU = 33
***Responsible advocacy*** Ensures resources are available to conduct TB clinical trials and ensures access as an element of the ethical goal of GPP‐TB. Includes consideration of the role of regulatory bodies and pharmaceutical companies, reduction in barriers and improved access when research is concluded	Summary score for responsible advocacy (range 0 to 5)	Identify stakeholders who are effective TB champions; provide educational briefings to policy makers	BL = 1 FU = 0	BL = 3 FU = 1	BL = 1 FU = 1
***Deliberation*** Ensures options for mutual gain are pursued when trade‐offs in GPP‐TB principles or benchmarks are needed. Entails formal discussions and negotiation between the various stakeholders who have a legitimate interest in the consequences that a trade‐off between considerations might have	Has a conflict between principles arisen? (Y/N)		BL = N FU = N	BL = Y FU = N	BL = N FU = N
	If Y: was there a structured opportunity where concerned stakeholders met? (Y/N)		BL = n/a FU = n/a	BL = N FU = n/a	BL = n/a FU = n/a
	If Y: summary score for deliberation process (range 0 to 6)	Explicit norms for discussion established; authority shared equally by all stakeholders	BL = n/a FU = n/a	BL = 0 FU = n/a	BL = n/a FU = n/a
	Ranked score for how site would respond to a future conflict (0 to 5)	PI would determine appropriate steps (0); research site would seek expert advice (2); conduct rapid assessment to map issues and who affected (5)	BL = 0 FU = 0	BL = 0 FU=0	BL = 2 FU = 0

Higher summary scores indicate more intensive use of practices associated with the strategy. n/a, not applicable.

#### 2.1.2 Outcome measures

Several short‐term outcome measures were included in the survey. The simplest measure was the total number of TB clinical trials implemented by the site. A set of three measures (mutual gain, transparency and integrity, shared knowledge) focused on the extent to which a site experienced specific challenges identified by the project advisory board. Scores for each measure were calculated based on whether the site reported that an item was not a challenge (1), somewhat of a challenge (−1) or a major challenge (−2). Mutual gain was calculated as the sum of responses to two challenges: competition with the public health system for human resources (i.e. qualified staff) and whether infrastructure built for TB trials uses standards relevant for the local health system. Transparency and integrity were scored on the response to the challenge of establishing effective communication networks for reporting and monitoring of TB cases identified. Shared knowledge was scored on the response to the challenge of ensuring local stakeholder understanding of TB disease, treatment and prevention. The final short‐term outcome measure was included in the Deliberation Strategy section of the survey and was specific to sites reporting that a conflict or tension had arisen in the last 12 months between research principles and/or principles of importance to other stakeholders in the local context. This was a ranked score item with a value of 2 if use of a structured opportunity led to a successful resolution, 1 if the issue was resolved through other means and 0 if a resolution was not reached.

One intermediate/long‐term outcome measure was included. This was a binary yes/no measure indicating whether the most recent clinical trial implemented by the site resulted in the experimental drug tested in the trial being available.

Three summary measures reflective of achieving the GPP‐TB ethical goal were included (Access, Social value and Acceptability). The summary measures were derived from responses to 13 potential outcomes for the most recent clinical trial implemented by the site (see Data S1 for a detailed breakdown). Each outcome was scored −1 if it indicated failure to meet the goal and +1 if it indicated success. Items were scored 0 if the site did not check it as a relevant outcome for the trial in question. Most items were reflective of more than one summary measure. Access was calculated as the sum of 11 responses (five negative, six positive) indicative of ability to successfully access study populations and complete the trial, for a successful drug to be accessible by providers and clients following the trial, and to contribute to better health outcomes nationally. Social value was calculated as the sum of five responses (one negative, four positive) indicative of ability to complete the trial, demonstrate efficacy, generate new TB treatment or prevention guidelines and contribute to better health outcomes nationally. Acceptability was calculated as the sum of five responses (three negative, two positive) indicative of suitability, availability, affordability and successful use of the drug tested in the trial for the local or national context.

### 2.2 Site recruitment

We worked with the CRAG to identify three geographically diverse CDC‐funded TBTC research sites willing to participate in the pre‐pilot. Participating sites were located in North America, South America and Asia. To preserve confidentiality, further details on site location are not provided here; the sites are designated as A, B and C without reference to geographic location. GPP‐TB guidelines explicitly state the importance of greater attention to the interests of stakeholders throughout the lifecycle of the research, including site selection, trial planning and site activation. We therefore included a site in the early stages of preparations for the conduct of TBTC‐sponsored clinical trials. We engaged research staff at each site in qualitative interviews (via phone, internet or in‐person), online surveys and training related to GPP‐TB. Participants at each site included staff engaged in TB trials‐related work including staff responsible for stakeholder/community engagement. Leadership at each of the three sites determined which staff were invited to participate in data collection.

### 2.3 Data collection and analysis

All data collection was conducted in the local language for each site. For quantitative data collection, we used self‐administered internet surveys (Qualtrics, Provo, UT, USA). Each site was asked to identify a point person who was contacted via email to provide the information needed for completion of the surveys. The email invitation included brief instructions and a link to the survey, which took about 30 minutes to complete. Because each survey could require knowledge or expertise shared by more than one person at a site, multiple staff may have helped to complete each survey. It was left up to each site to determine how and by whom each survey was completed.

Because of its recent development, sites had little or no exposure to GPP‐TB. We therefore developed a three‐part introductory training on GPP‐TB (about four hours duration total), conducted via internet (North America and South America) or onsite (Asia) after baseline data collection was completed. The GPP‐TB training was open to TBTC site staff, whether they participated in the data collection or not, at the discretion of site leadership. Trainings were conducted in the local language for each site.

Approximately six months after the training, each site completed a follow‐up survey using the same measures as at baseline. While this timeframe was too short to fully pilot our ability to track significant impacts of GPP‐TB implementation on ethical outcomes, it provided additional insights into variability within and between sites, which is helpful for informing further development of a rigorous evaluation design.

Given the small number of sites, we used simple frequencies to identify the strategies, practices and outcomes identified in the TOC framework. We looked at similarities and differences between the three sites as well as changes from baseline to follow‐up within sites. We used reporting functions within Qualtrics and Excel for the descriptive analysis.

The qualitative interviews were conducted via phone with the North and South America sites, and in‐person at the Asia site. Interviews were recorded using digital audio recorders combined with note taking; recordings were transcribed verbatim, translated into English (where necessary) and supplemented with the notes. The interviews were conducted individually for the North America (n = 3) and Asia (n = 5) sites and in a small group interview with three site staff along with a separate individual interview (total n = 4) in South America. As with the surveys, it was left up to each site to determine with whom the interviews were conducted. At a minimum, we requested participation of an investigator, community outreach staff or CAB representative, and a study manager. Transcripts were analysed using a structural coding framework that reflected awareness of the core elements of GPP‐TB (levels of stakeholders, principles, benchmarks and steps or practices outlined for stakeholder engagement) and the elements of the TOC framework (strategies, outcomes and ethical goal).

## 3 Results

### 3.1 Survey findings

#### 3.1.1 Powerful strategies

We observed variability in the survey measures of powerful strategies as well as room for both increases and decreases in the intensity of use of practices reflective of each strategy (Table 1). For example, under *Accountability*, two sites reported having community advisory boards (CABs) at baseline, one reporting 19 and the other 25 CAB‐specific practices out of 60 potential practices. Examples of CAB‐specific practices included how CAB members were identified (e.g. recruitment targeted to maximize diversity, community leaders or TB patients asked to recommend members), frequency of CAB meetings and of participation by research team members other than community liaison/outreach staff, and types of resources provided to support CABs (e.g. meeting supplies such as paper and pens, computer/internet access for members, transportation support). The site without a CAB reported the highest number of general engagement practices (n = 14) out of a total of 43 potential practices. Examples of general engagement practices included meetings with community stakeholders, health education events, research literacy training and engagement with stakeholders to discuss mobilization, sensitization or education related to trials. At follow‐up, Site A no longer reported having a CAB or using general engagement strategies.

Site B reported the only instance of a conflict requiring *Deliberation* strategies to balance competing principles, at baseline. No structured opportunity was provided for concerned stakeholders to meet and the site reported that the conflict was not successfully resolved. With the exception of Site C at baseline, the three sites reported that the principal investigator (PI) would determine appropriate steps to respond, should a future conflict arise, indicating minimal to no community/stakeholder engagement strategies in place should a controversy escalate.

Sites reported similar intensity of practices related to *Community Mapping* and *Shared Learning* strategies at both baseline and follow‐up. There was some variability in *Responsible Advocacy* practices, with Site B reporting the most use of such practices at baseline.

#### 3.1.2 Outcomes

Responses to the survey questions on outcomes are summarized in Table 2. The three participating sites had a range of experience conducting TB clinical trials. Site A reported conducting seven trials at baseline and eight at follow‐up, Site B reported three at baseline and six at follow‐up, and Site C (a recently funded TBTC trial site at the time of data collection) reported no trials at baseline or follow‐up. We did not ask sites to identify the specific trials that were reported on in the baseline and follow‐up surveys, and it is possible that one or both sites may have reported on the same trial in both surveys.

**Table 2:** Baseline (BL) and follow‐up (FU) responses to survey outcome measures at three sites

Type of outcome	Indicators	Examples of items included in scores	Site A	Site B	Site C
Short term	Ranked score for conflict outcome (0 to 2)	Use of a structured opportunity for deliberation led to successful resolution (2); no structured opportunity but resolved through other means (1); unable to reach agreement (0)	BL = n/a FU = n/a	BL = 0 FU = n/a	BL = n/a FU = n/a
	Total number of TB clinical trials implemented		BL = 7 FU = 8	BL = 3 FU = 6	BL = 0 FU = 0
	Mutual gain challenges (−4 to 2)	Competition with the public health system for human resources (i.e. qualified staff); infrastructure built for TB trials uses standards relevant for the local health system	BL = 2 FU = −1	BL = −2 FU = −3	BL = 0 FU = 0
	Transparency and integrity challenges (−2 to 1)	Establishing effective communication networks for reporting monitoring of TB cases identified	BL = 1 FU = −1	BL = −2 FU = −1	BL = 1 FU = −1
	Shared knowledge challenges (−2 to 1)	Ensuring local stakeholder understanding of TB disease, treatment and prevention	BL = 1 FU = −1	BL = 1 FU = −1	BL = 1 FU=.
Intermediate & long term	Effective product available as result of most recent trial (Y/N)		BL = Y FU = Y	BL = N FU = Y	n/a
GPP‐TB goal	Access summary score for most recent trial (−5 to 6)	Our site was not able to recruit the target number of participants (−1); the experimental drug tested in the trial is not suitable for use in the local context (−1); the experimental drug tested in the trial is available (1)	BL = 4 FU = 3	BL = −1 FU = 1	n/a
	Social value summary score for most recent trial (−1 to 4)	The trial was closed early (−1); the trial was successfully completed (1); the trial ultimately led to new TB treatment or prevention guidelines (1)	BL = 4 FU = 4	BL = 1 FU = 1	n/a
	Acceptability summary score for most recent trial (−3 to 2)	The experimental drug tested in the trial is not suitable for use in the local context (−1); the experimental drug tested in the trial is available but many providers refuse to use it (−1); the experimental drug tested in the trial is available and successfully used by providers and patients (1)	BL = 2 FU = 2	BL = 0 FU = 0	n/a

. , missing value.

### 3.2 Qualitative findings

Analysis of the qualitative interviews was informative about how sites conceptualized the concepts underlying the participatory strategies, the extent to which baseline practices were aligned with the TOC framing for each and the extent to which they viewed their work as aligned with the elements of the GPP‐TB goal statement. As a reminder, interviews were conducted before the baseline survey and GPP‐TB training for each site. Interviews indicated low familiarity with GPP‐TB across all sites and confusion with Good Clinical Practices (GCP) was common.

Regarding *accountability mechanisms*, questions about who would be considered a TB trial stakeholder, and how information would (or would not) be shared with them, elicited responses focused primarily on three dimensions. Firstly, they described the complex relationships within research groups (investigators, protocol teams, sponsors, laboratories, regulatory groups, etc.). Secondly, they noted the importance of the relationship between patient‐participants and clinician‐researchers due to the highly burdensome nature of trial requirements (e.g. daily observed therapy, dietary requirements). Thirdly, they discussed the importance of relationship building between researchers and health system providers to facilitate access to patient populations for trial participation. Accountability questions prompted reflections on GCP with little reference to research participants and their communities. Also of note, Site A reported no CAB in the qualitative interview, although reported one in the survey at baseline.

*Community mapping* as a strategy was described as reliant primarily on local health departments and clinics as sources of data, such as disease trends in subpopulations or areas, and on the personal knowledge of research staff regarding issues impacting the community, such as ease of access to health care or economic stresses impacting patients. One site reported their staff visited clinics to better understand “…how drugs are distributed for patients; patients come to the health station for taking drugs or health workers provide drugs at their home, we want to know about the distance between their house and [the clinic].” Journal clubs, presentations, seminars, trainings and conference attendance were mentioned as mechanisms for research staff to keep up with public health issues related to TB more broadly, but as one site noted, “we do it to some extent, but probably could do more.” Another site noted “there is no budget for this, we know it is important.”

Discussion of *shared learning* as a strategy focused on working with stakeholders individually or in small groups to share information considered of most value to them, for example, targeted information for patients enrolled in research, TBTC collaborators, health department TB clinic staff, laboratory technicians and nongovernmental organizations addressing TB in the community. Mechanisms for information sharing with the affected community more broadly were generally associated with events like World TB Day and focused on TB generally, with minimal or no attention to a site's research agenda. Limited staffing and budgets were noted as barriers to more systematic information sharing, with most effort going towards working one‐on‐one to support study participants. While the importance of broader community engagement was noted by each site, the “how and why” of information sharing with community stakeholders was not clearly articulated.

Use of *responsible advocacy* as a strategy was limited. One site focused around World TB Day activities, with participation and support by research staff but not leadership for the events. All sites described advocacy primarily to gain support from TB treatment programmes for referral of patients to clinical trials. One site described a recent medical research controversy precipitated by a very critical newspaper article (not TB related), noting “This article has caused a lot of damage for the research community in our country” but also:

…in part, this [controversy] is the researchers’ wrong doing as [education] is only done in response to a negative media publication or communication instead of being consistent and trying to use the communication/media to work on our side so the researchers are taken serious and not how it is described in the media.

To understand how *deliberation* was or might be used at the sites, we asked first if the site had faced any research‐related dilemmas that required finding a balance between competing principles or values. If yes, we asked for a description of the dilemma and its resolution. If not, we asked sites to think about a situation where such a dilemma might arise and how their site would likely resolve it. We then asked how typical the approach was, whether there were dilemmas that might require a different approach and what options might be used in the case of stalemate or deadlock on a resolution. Types of dilemmas centred on balancing the needs of participants with study requirements, for example, issues of stigma, addressing patient fears about research, delays in starting treatment due to study requirements for preliminary testing and whether treatment for another illness could be modified so that a patient could qualify for a TB trial. In all cases, hypothesized or real, sites emphasized the importance of a “team effort” for resolving dilemmas, which could potentially include community stakeholders, patients and their family members, and research staff. However, when asked how the site would deal with a stalemate, all sites indicated that the PI would have the final say in how the dilemma would be resolved.

In discussions about the elements of the *GPP‐TB goal statement*, social value centred on local responsiveness and getting a good match between a research study and patient population needs. One site noted, “There have been a couple of trials that we haven't participated in directly, because they just didn't seem to be very relevant to the population of our TB patients…so the main emphasis is on, is it going to be clinically relevant for our practice here? But of course we hope to be able to make some contributions to improving the global TB care.” Another site noted multiple benefits of research, including “improved community awareness and shortening the TB treatment period; a second benefit is TBTC sites have been restructured and equipment has been provided with funding support from the donor, [and] capability of health workers also improved.”

When asked how much consideration sites gave to whether a trial drug was something providers in their location would prescribe, one site noted the combined considerations of cost and funding: “We don't know if it will be accessible to the community and we have to trust the [pharmaceutical company] to take this into consideration…Unfortunately this is also a political issue in our country.” Post‐trial access to effective drugs was viewed as very important by all sites, although viewpoints varied regarding the relative influence or role of regulatory agencies, providers and pharmaceutical companies in assuring such access; the potential role of advocates or civil society did not come up. Sites did not feel strongly empowered to influence funders and regulatory agencies, but rather saw their role as more passive and subject to the direction from others, for example, “We can [try to] persuade policy makers but we won't achieve success every time” and “We can only suggest, we can start the conversation with the entities that make these decisions but we cannot put pressure on them, it will not guarantee the approval.”

There was general agreement that all stakeholders, including patients, should have access to the research results, although sites were sensitive to confidentiality issues related to how patients were re‐contacted to share results.

## 4 Discussion

Evaluation efforts have not kept pace with the expanding calls for greater use of GPP and other participatory engagement models in HIV, TB and other challenging clinical research contexts 3, 4, 33, 34, 35, 36. Limited empirical data exist on the contribution of GPP to clinical trials or even descriptive data on what clinical trial sites are doing when they implement GPP. Outcome evaluation of GPP as a global endeavour is a complex problem that has not received any attention. The study presented here is a first and basic, but essential, step towards building an outcome evaluation framework for GPP and related participatory models for clinical trials. The study demonstrated the feasibility of collecting informative data aligned with elements of a TOC evaluation framework and using a minimally burdensome online survey in multiple languages. The measures captured considerable variability in the intensity and variety of engagement strategies, both across and within sites over time. Sites were forthcoming regarding selected outcomes reflective of the GPP‐TB goal statement.

In developing the measures, we were keenly aware of the need to generate a descriptive baseline of strategies and practices to gain meaningful insight into what works and under what conditions. The strategies and practices in our TOC framework reflect the purposeful framing and selection of a broad universe of engagement strategies and practices that the developers of the TOC framework believe will lead to the desired outcomes and goal of GPP‐TB. Trial sites may be using many of these existing practices without reference to any of the GPP guidance documents, including sites that are new to clinical trials research if they already have a culture of engaged community practice in other work. Conversely, even experienced trial sites with knowledge of GPP may not be using some, or any of the strategies included in our TOC framework.

Generating a baseline description of engagement practices in use and not in use by trial sites is a necessary step in ultimately being able to evaluate the contribution of intentional strategies and practices to long‐term desired outcomes. In this regard, it is helpful to think of GPP as a widely used intervention to improve ethical, social and scientific outcomes of clinical trials that is not fully standardized and has not been evaluated for effectiveness. Establishing a baseline description of what is and is not being done in the name of GPP is a basic requirement to move the field of practice forward on something stronger than anecdotal evidence. The strategy measures developed for this study, while not comprehensive for all engagement models, are likely to have broader applicability than the evaluation context of GPP‐TB. For example, our intentional inclusion of non‐CAB engagement practices as part of the *Accountability* strategy reflects calls by others of the need for broader mechanisms of community and stakeholder engagement 35. There is also clear benefit to be gained from exploring how the GPP‐TB TOC framework measures align with others being developed within the broader field of community‐based participatory research 37.

The small number of sites included in this pre‐pilot makes it difficult to identify meaningful patterns in the data, and such an analysis was not one of the objectives of this study. That said, one interesting point is the fact that Site B used several *Responsible Advocacy* practices in the same time period that they were unsuccessfully struggling to address a conflict in need of *Deliberation*, and had been unable to recruit the target number of participants for the most recent TB trial conducted at the site. Regarding *Accountability Mechanisms*, the site had no CAB but reported more general engagement practices than the other sites, and, in the six months following the baseline survey, they reported three additional trials being conducted. The ability to parse such patterns with data from only three sites is promising for more rigorous analysis with more robust data, and for generating potentially testable hypotheses, for example, in line with a Realist Evaluation approach to determining what works, for whom and under what conditions 38, 39.

The qualitative data added rich detail about the way research staff who were largely unfamiliar with GPP‐TB perceived the strategies, practices and outcomes outlined in the TOC framework. At times, site staff did not understand the questions and asked for clarification, said they could not answer the question, or responded with information derived from GCP guidelines or local regulatory requirements. The fallback to GCP is not surprising, given the emphasis on training and compliance with GCP for trial sites. But it underscores the importance of building a shared lexicon around the basic concepts and principles of engagement, to ensure that all stakeholders inclusive of trial staff do not talk past each other. It is encouraging to note that endorsement of the core elements of the GPP‐TB goal statement was evident across all three sites.

Lessons learned from this pre‐pilot point to several challenges for implementing a more comprehensive evaluation of GPP‐TB (or other engagement models) aligned with a TOC framework. First, this was a small pre‐pilot study with three sites with limited generalizability; a more comprehensive global survey process would require more extensive work to build support among clinical trialists and demonstrate the value of the resulting data for their practice as researchers. Second, the survey responses were self‐reported data, and may be subject to the various forms of misreporting generally associated with self‐reported data. For example, Site A reported having a CAB in the baseline survey but indicated no CAB present at their site during the qualitative interviews conducted around the same time. This may have been due to differing interpretations of what a CAB is, including whether the CAB needs to be specific to a research site or could reference an advisory board whose members are drawn from multiple communities participating in trials sponsored by a network such as the CDC TBTC. Additional testing is needed to ensure the measures used are valid and robust, especially when translated into multiple languages. Third, a limited set of outcomes reflective of the TOC framework were measured and all were subject to self‐report bias. Outcome measures could potentially be collected through more objective means, such as online clinical trial registries, peer‐review publications, treatment guidelines and recommendations, and epidemiological reports on disease trends. Fourth, additional measures such as stakeholder understanding of potential trial outcomes, the extent of shared knowledge and perceptions of transparency and integrity require data collection with stakeholders beyond the research team to understand how they perceive and experience the changes hypothesized to result from the use of the strategies and practices. Such measurement presents additional challenges for recruiting participants and data collection in settings where stakeholders are likely to be geographically dispersed, linguistically diverse, with a range of literacy, and potentially limited ability to respond to an online survey.

## 5 Conclusions

Community and stakeholder engagement in clinical trials for HIV, its comorbidities and other socially complex diseases is recognized as of value both ethically and practically. But systematic efforts to evaluate what works, for whom and under what conditions in the context of TB and other clinical trials are lacking. Results from this exploratory pre‐pilot indicate the feasibility of generating a description of the variety and intensity of engagement practices being used by research sites globally. Capturing such variability is a necessary step for assessing how particular strategies and practices correlate with desired outcomes (such as timely recruitment, retention and uptake of results) and, potentially, how well they predict such outcomes when observed at multiple sites over time. This type of global survey would be a valuable addition to building a theory‐driven, mixed methods evaluation approach to better understand and enhance engagement as a critical component of global clinical research.

## Competing interests

KM reports a grant from the National Institutes of Health during the conduct of the study; personal fees and grants from the National Institutes of Health and grants from the United States Agency for International Development and the FHI Foundation outside of the submitted work. NE, MF and CH report work under a grant from the National Institutes of Health during the conduct of the study. NE and CH report work under a grant from the United States Agency for International Development outside of the submitted work. MF reports that his employer (Treatment Action Group) received a grant from the Veterans Health Administration for activities with the Tuberculosis Trials Consortium outside of the submitted work. CH reports longstanding funding through the Veterans Health Administration for TBTC work.

## Authors’ contributions

KM, NE, MF and CH contributed to conception and design of the study and interpretation of the data. KM and NE contributed to data collection and analysis. KM wrote the manuscript and NE, MF and CH contributed to critical revisions. All authors contributed to the writing of the manuscript and reviewed and approved the final version.

## Supporting information


**Data S1.** Survey indicators and measures, including reference to the Theory of Change framework components, the rationale for each indicator measure, the range of values associated with indicators, the wording of questions for each measure, how values were calculated from responses, and the response items within measures.
