# Supplementary material for: Using theory of change frameworks to develop evaluation strategies for research engagement: results of a pre‐pilot study
Source: J Int AIDS Soc. 2018 Oct 18;21(Suppl Suppl 7):e25181. doi: 10.1002/jia2.25181 (PMC6193313; doi:10.1002/jia2.25181)
Supplement: Supplementary file 1 — Data S1. Survey indicators and measures, including reference to the Theory of Change framework components, the rationale for each indicator measure, the range of values associated with indicators, the wording of questions for each measure, how values were calculated from responses, and the response items within measures. [file JIA2-21-e25181-s001.pdf]

For information on the Theory of Change framework see:
MacQueen, K. M., et al. (2016). "Developing a Framework for Evaluating Ethical Outcomes of Good Participatory Practices in TB Clinical Drug Trials." J Empir Res Hum Res Ethics **11**(3): 203-213. DOI: 10.1177/1556264616657452

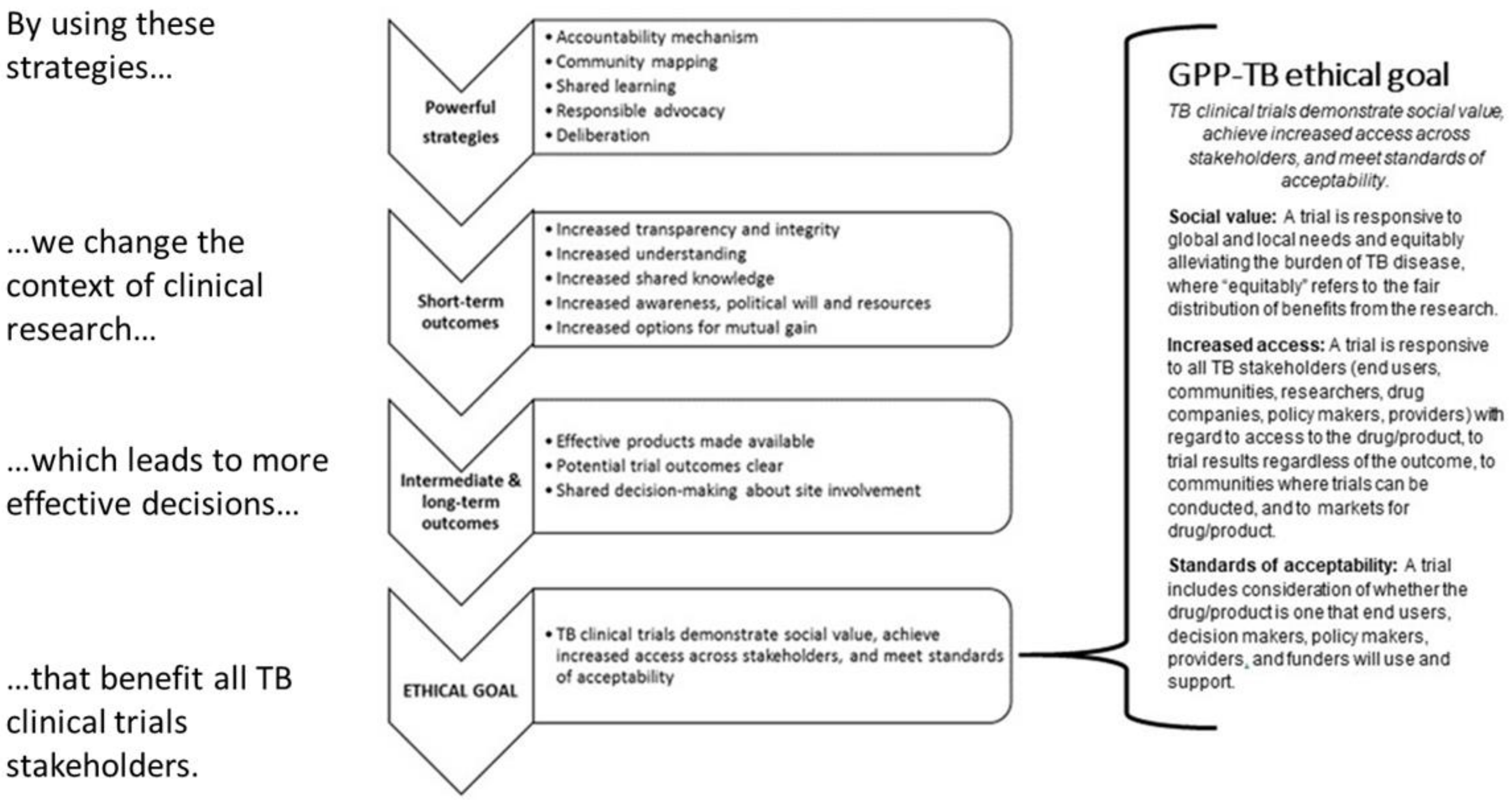

Figure 1. Theory of Change framework for evaluating Good Participatory Practices for TB Clinical Trials. A set of powerful strategies, each comprised of a range of potential practices, are hypothesized to lead to short-term, intermediate, and long-term outcomes that cumulatively result in achieving the elements outlined in the GPP-TB ethical goal. To qualify as powerful, a convincing argument or causal hypothesis had to be made for how a proposed strategy would lead to outcomes that in turn would lead to achieving the ethical goal.

For additional information contact Kate MacQueen kmacqueen@fhi360.org *or Natalie Eley* neley@fhi360.org

Table 1. Survey indicators and measures, including reference to the Theory of Change framework components, the rationale for each indicator measure, the range of values associated with indicators, the wording of questions for each measure, how values were calculated from responses, and the response items within measures.

TOC Framework component	Indicators	Sub-measures within indicators<br>*Note: includes questions as worded in survey & logic for calculating value based on responses*	Response items within measures
**Powerful strategies**<br>*Note: each strategy color coded in this column*			
Accountability mechanisms	[Non-indicator item]	Q2.1 The first set of questions are about accountability mechanisms. Accountability mechanisms can be used to increase transparency and integrity in representing individual and stakeholder group interests and expectations, and in maintaining scientific and ethical standards.	
	CAB presence<br>• Rationale: presence of a CAB indicates an important accountability mechanism<br>• Range: 0-1	Q2.2 Does your site have an active community advisory board (CAB), community advisory group (CAG) or similar mechanism in place for TB-related research? By active we mean that the board or group has met formally with research staff at least once in the past 12 months.<br>• If (1) selected then value=1<br>• If (2) selected then value=0	(1) Yes<br>(2) No
		NOTE: If YES to CAB presence question above then complete following questions; else skip to Non-CAB Engagement (Q2.13).	
	[Non-indicator question]	Q2.3 What do you call your CAB-type mechanism?	
	CAB research diversity<br>• Rationale: higher value indicates greater diversity of stakeholder perspectives related to TB in CAB<br>• Range: 1-3	Q2.4 Does your CAB-type mechanism provide support only for TB-related research or for other purposes as well?<br>• If (1) or (4) selected then value=1<br>• If (2) or (5) selected then value=2<br>• If (3) or (6) selected then value=3<br>• If (7) then value=1	(1) TB-related research only<br>(2) TB and HIV-related research only<br>(3) A range of research activities, not limited to TB or HIV<br>(4) TB-related research and programs<br>(5) TB and HIV-related research and programs<br>(6) A range of research and program activities, not limited to TB or HIV<br>(7) Other, please describe
	CAB age<br>• Rationale: higher score indicates greater sustainability of CAB mechanism	Q2.5 How long has your CAB-type mechanism been in existence?<br>• If (1) then value=1<br>• If (2) then value=2<br>• If (3) then value=3	(1) One year or less<br>(2) 2-3 years<br>(3) 4-5 years<br>(4) More than 5 years

**TOC Framework component**	**Indicators**	**Sub-measures within indicators**<br>*Note: includes questions as worded in survey & logic for calculating value based on responses*	**Response items within measures**
	• Range: 1-4	• If (4) then value=4	
	CAB outreach<br>• Rationale: higher score indicates greater effort to enhance inclusion of diverse stakeholders<br>• Range: 0 to 10	Q2.6 Which of the following best describe how your CAB-type mechanism was created? Please check all that apply.<br>• Value=sum of total number of items checked	• We recruited members to maximize diversity, e.g. race, color, religion, gender, sexual orientation, ethnicity, age, disability, socio-economic status, etc.<br>• We recruited members to reflect diversity in professional or personal experience, e.g. people living with HIV/AIDS, current. or former research participants, representatives from community-based organizations<br>• We asked community leaders to recommend people as members<br>• We asked TB patients to recommend people as members<br>• We asked current or former research participants to recommend people as members<br>• We asked current members to recommend other people as members<br>• We asked local TB and/or HIV advocates to recommend people as members<br>• We advertised for volunteers, e.g., by posting flyers or sending open invitations to a variety of organizations<br>• We recruited people in key leadership positions to be members, e.g., from government offices, educational institutions, religious organizations, traditional leaders<br>• Other: please describe

**TOC Framework component**	**Indicators**	**Sub-measures within indicators**<br>*Note: includes questions as worded in survey & logic for calculating value based on responses*	**Response items within measures**
	CAB members<br>• Rationale: higher score indicates greater diversity of stakeholders on CAB<br>• Range: 0-9	Q2.7 Thinking about the currently active TB research participants members of your site's CAB-type mechanism, how would you describe them? Please check all that apply.<br>• Value=sum of total number of items checked	• TB patients, their families, partners, neighbors or co-workers<br>• TB advocates<br>• HIV advocates<br>• Other local community members (not directly impacted by TB)<br>• Health care program and service providers<br>• Policy makers<br>• Researchers, funding agency representatives or regulatory body representatives<br>• Research staff, e.g., study coordinator, community liaison officer, outreach worker, counselor<br>• Other, please describe
	CAB size rationale:<br>• Larger size indicates greater general inclusion of stakeholders<br>○ Range: 1-4<br><br>CAB tenure rationale:<br>• Longer average tenure indicates more stable CAB membership<br>○ Range: 1-5	Q2.8 We would like to know how long current members of your CAB-type mechanism have been active. Please use the sliding bar to indicate the number of members active for each length of time indicated, from 0 to 10 or more years.<br>• CAB size: sum number of CAB members in each duration category<br>○ If sum <6 then value=1<br>○ If sum >5 and <11 then value=2<br>○ If sum >10 and <21 then value =3<br>○ If sum >20 then value=4<br>• CAB tenure: value=sum of (number of CAB members in duration category x the value for that category); divide by value for CAB size and round to nearest whole number	(1) One year or less<br>(2) 2-3 years<br>(3) 4-5 years<br>(4) 6-10 years<br>(5) More than 10 years<br><br>*NOTE: Use of the Qualtrics sliding bar response option proved problematic for this question; a simpler framing is needed.*
	CAB meeting frequency<br>• Rationale: higher number indicates greater level of interaction<br>• Range: 1-6	Q2.9 How often does your site's CAB-type mechanism meet?<br>• If (1) then value=1<br>• If (2) then value=2<br>• If (3) then value=3<br>• If (4) then value=4<br>• If (5) then value=5<br>• If (6) then value=6	(1) About once a year<br>(2) About twice per year (every 6 months)<br>(3) About 3 times per year (every 4 months)<br>(4) About 4 times per year (quarterly or every 3 months)<br>(5) About every 2 months<br>(6) About every month

**TOC Framework component**	**Indicators**	**Sub-measures within indicators**<br>*Note: includes questions as worded in survey & logic for calculating value based on responses*	**Response items within measures**
	CAB frequency of interaction with research staff<br>• Rationale: higher number indicates greater level of interaction<br>• Range: 1-4	Q2.10 How often do meetings of your site's CAB-type mechanism include participation by research team members other than community liaison/outreach/education staff?<br>• If (1) then value=1<br>• If (2) then value=2<br>• If (3) then value=3<br>• If (4) then value=4	(1) Rarely or never<br>(2) Occasionally<br>(3) Most meetings<br>(4) Every meeting
	CAB documentation<br>• Rationale: higher number indicates more tools for transparency and accountability<br>• Range: 0-9	Q2.11 Which of the following documentation does your site typically keep for your CAB-type mechanism? Please check all that apply.<br>• Value=sum of checked items	• Meeting agenda<br>• Attendance record<br>• Meeting minutes<br>• Budget line item(s) and tracking of expenditures<br>• Member orientation or training sessions<br>• Membership roster<br>• Group charter<br>• Dedicated staff time outlined in one or more position descriptions<br>• Inclusion of CAB-type mechanism in investigator workplan
	CAB resources<br>• Rationale: higher number indicates better support for CAB functioning<br>• Range: 0-6	Q2.12 Does your site provide the following resources to support your CAB-type mechanism? Please check all that apply.<br>• Value=sum of checked items	• Physical space for meetings<br>• Meeting supplies, e.g., paper, pens, markers<br>• Computer and internet access for members<br>• Refreshments during meetings<br>• Transportation support for members attending meetings<br>• Honoraria or other payment for members
	Intensity score for CAB-specific practices<br>• Rationale: greater use of a variety of CAB-specific practices indicates greater transparency and accountability<br>Range: 6-60	• Value=sum of values for CAB-specific practices (Q2.4-Q2.12)	
		NOTE: Sub-measures below answered by all sites, regardless of response to CAB question (Q2.2).	

**TOC Framework component**	**Indicators**	**Sub-measures within indicators**<br>*Note: includes questions as worded in survey & logic for calculating value based on responses*	**Response items within measures**
	Engagement tools<br>• Rationale: greater diversity of engagement mechanisms indicates better outreach and transparency<br>• Range: 0-17	Q2.13 There are many ways that a research site may engage communities and stakeholders, in addition to using a CAB-type mechanism. In the following list please check each mechanism that has been used by your site at least once in the last 12 months.<br>• If (18) checked then value=0; Else value=sum of checked items	• One-to-one meetings with community members/stakeholders<br>• Routine/scheduled meeting with a community group or organization other than CAB-type mechanism<br>• Unscheduled or emergency meeting with a community group or organization other than CAB-type mechanism<br>• Health education event (general)<br>• Health screening event<br>• Health worker training<br>• Research literacy training<br>• Theatre/music event<br>• Sport event<br>• Radio, television or print advertisement<br>• Radio, television or print interview<br>• Press release<br>• Social media update, e.g., Facebook, Twitter<br>• Internet platform, e.g., website<br>• Posters or flyers<br>• Billboard<br>• Other, please describe<br>(18) No non-CAB-type engagement mechanisms used in last 12 months

**TOC Framework component**	**Indicators**	**Sub-measures within indicators**<br>*Note: includes questions as worded in survey & logic for calculating value based on responses*	**Response items within measures**
	Engagement topics<br>• Rationale: Coverage of more issues or topics indicates better outreach and transparency<br>• Range: 0-18	Q2.14 Thinking about all the ways that your site has engaged with community members and stakeholders, including CAB-type mechanisms, which of the following issues or topics were addressed in the last 12 months? Please check all that apply.<br>• If (19) checked then value=0; Else value=sum of checked items	• Protocol development and review<br>• Site selection for trial<br>• Resource identification<br>• Facilitating community entry or introductions<br>• Communications strategies<br>• Study briefing or information updates<br>• Stakeholder advisory or education plans<br>• Training<br>• Formative work needed to plan for trials<br>• Mobilization, sensitization or education related to trials<br>• Issues management plans<br>• Trial recruitment<br>• Informed consent<br>• Retention issues<br>• Adherence<br>• Results dissemination<br>• Post-trial access to care<br>• Other: please describe<br>(19) No engagement with community/stakeholders in previous 12 months
	Engagement language and format<br>• Rationale: Use of stakeholders' preferred language and format indicates greater transparency<br>• Range: 0-3	Q2.15 Thinking about all the different community members and stakeholders that your site has interacted with in the last 12 months, how often was your site able to provide updates to them in their preferred language and format (e.g., face-to-face meetings, newsletters, email)?<br>• If (1) then value=0<br>• If (2) then value=1<br>• If (3) then value=2<br>• If (4) then value=3	(1) Our site has not provided any updates to community members and stakeholder in the last 12 months<br>(2) We are rarely able to provide updates in the preferred language and format<br>(3) We are usually able to provide updates in the preferred language and format<br>(4) We always provide updates in the preferred language and format
	Engagement log<br>• Rationale: systematic documentation indicates greater transparency and accountability<br>• Range: 0-1	Q2.16 Does your site maintain an up to date log of communications and interactions with community members and stakeholders? This could be a spreadsheet, data entry system or paper file that records when the communication or interaction happened, who	(1) Yes<br>(2) No

**TOC Framework component**	**Indicators**	**Sub-measures within indicators**<br>*Note: includes questions as worded in survey & logic for calculating value based on responses*	**Response items within measures**
		was involved, what was discussed and whether any follow up was required.<br>• If (1) then value=1<br>• If (2) then value=0	
	Engagement specific to research events<br>• Rationale: More engagement activities associated with important research-related events indicates greater transparency and accountability<br>• Range: 0-4	Q2.17 For each of the following, please indicate whether this is something your site has experienced in the last 12 months and, if so, whether any community/stakeholder engagement occurred related to that event.<br>• If no event checked then value=0<br>• Else value=sum [total number of types of events checked] plus [total number of engagement activities checked] divided by total number of types of events; round to nearest whole number	Type of events experienced; check all that apply<br>• Submission of a new protocol to the local ethics committee<br>• Failure to obtain the necessary approvals to implement the research<br>• Research withdrawn following approval but prior to activation<br>• Study activation and enrollment of first trial participant<br>• Study is suspended or a suspension is lifted<br>• Study closure<br>• Publication or presentation of trial outcomes<br><br>Engagement activities; for each event checked above, check activities that apply<br>• CAB-type meeting held<br>• Community forum held<br>• Other dissemination done
	Intensity score for Non-CAB engagement<br>• Rationale: greater use of a variety of non-CAB practices indicates greater transparency and accountability<br>Range: 0-43	• Value=sum of values for Non-CAB engagement practices (Q2.13-Q2.17)	
Community mapping	[Non-indicator item]	Q3.1 The next set of questions are about community mapping. Community mapping is a strategy that research sites can use to identify stakeholder needs and improve understanding of context with regard to the local community, the TB research community, and the global public health community.	

**TOC Framework component**	**Indicators**	**Sub-measures within indicators**<br>*Note: includes questions as worded in survey & logic for calculating value based on responses*	**Response items within measures**
	Map locations<br>• Rationale: access to geographic maps indicates greater access to information about local context.<br>• Range: 0-3	Q3.2 Do research staff at your site have access to one or more geographic maps showing the following? Please check all that apply.<br>• Value=sum of checked items	• Where potential or enrolled research participants reside, labeled using locally meaningful designations<br>• Clinic locations where research participants can obtain services outside of the research setting<br>• Transportation options for participants to get to the research clinic and referral sites
	Map sociodemographics and epidemiology<br>• Rationale: access to current sociodemographics and epidemiology indicates greater knowledge of local context<br>• Range: 0-1	Q3.3 Can research staff at your site readily access current sources of information on sociodemographic and health characteristics of the communities where potential or enrolled research participants reside?<br>• If (1) then value=1<br>• If (2) then value=0	(1) Yes<br>(2) No
	Map leadership<br>• Rationale: more systematic tracking of local leadership indicates greater knowledge of local context<br>• Range: 0-2	Q3.4 Can research staff at your site readily identify current local elected officials and traditional leaders in the communities where potential or enrolled research participants reside?<br>• If (1) then value=2<br>• If (2) then value=0<br>• If (3) then value=1	(1) Yes, our site maintains a database or documentation with this information<br>(2) No, our site does not systematically track this information<br>(3) Uncertain; some staff may have this information readily available but we do not systematically track it

**TOC Framework component**	**Indicators**	**Sub-measures within indicators**<br>*Note: includes questions as worded in survey & logic for calculating value based on responses*	**Response items within measures**
	Map documentation of stakeholder information<br>• Rationale: more systematic documentation of information about stakeholders indicates greater knowledge of context<br>• Range: 0-21	Q3.5 The following is a list of types of stakeholders. Please indicate whether research staff at your site has documented information on groups and organizations representing each stakeholder group, the priority interests of concerns of each group, and each group's perspectives about TB research.<br>• Value=total number of items checked (maximum 7 types of stakeholders x 3 types of documentation = 21)	Types of stakeholders:<br>• TB patients, their families, partners, neighbors or co-workers<br>• TB advocates<br>• Other local community members (not directly impacted by TB)<br>• Health care program and service providers<br>• Policy makers<br>• Researchers, funding agency representatives or regulatory body representatives<br><br>For each type of stakeholder, check if your site has documented information about (check all that apply):<br>• Groups and organizations<br>• Priority interests and concerns<br>• Perspectives about TB research
	Map participant experience<br>• Rationale: Interviews with participants about their research experience indicates greater knowledge<br>• Range: 0-1	Q3.6 In the last 12 months has your research site conducted interviews with TB trial participants to understand their experience as research participants?<br>• If (1) then value=0<br>• If (2) then value=1<br>• If (3) then value=missing	(1) No, we have not conducted such interviews with TB trial participants<br>(2) Yes, we conducted interviews TB trial participants to understand their experience<br>(3) Our site has not conducted any TB trials in the last 12 months
	Map information sharing<br>• Rationale: Greater availability of information about people responsible for the researcher increases understanding of research by community members and stakeholders<br>• Range: 0-5	Q3.7 In thinking about the TB trials research your site has done in the last 12 months please indicate which of the following information your site would make freely available to community members and stakeholders. Please check all that apply.<br>• If (6) checked then value=missing<br>• Else value=sum of checked items	• A listing of key local personnel associated with each trial that includes name, job title, role, and responsibilities<br>• A description of the formal approval process for trial implementation and continuation that explains the kind of review done at each step, who is

TOC Framework component	Indicators	Sub-measures within indicators<br>*Note: includes questions as worded in survey & logic for calculating value based on responses*	Response items within measures
			responsible for doing that review, and the extent of their regulatory authority<br>• Information about all trial funders and sponsors<br>• Information about other trial partners such as laboratories or other research sites<br>• A description of the formal approval process needed to make a drug available for use in-country, if a trial indicates it is effective<br>(6) Our site has not conducted any TB trials in the last 12 months
	Map global relations<br>• Rationale: Relationships with global stakeholders indicate greater understanding of global context of research<br>• Range: 0-3	Q3.8 Are there research staff at your site who have established relationships with the following? Please check all that apply.<br>• Value=sum of checked items	• Sponsors and funders of proposed, on-going and completed TB trials in your country<br>• International advocacy groups that have an interest in supporting TB trials research and/or communities affected by TB<br>• Normative bodies (e.g., WHO, CDC) that provide guidance related to TB and related health issues
	Map global debates<br>• Rationale: Tracking of debates indicates greater understanding of global context of research<br>• Range: 0-2	Q3.9 To what extent does your research site track global debates with relevance to specific TB trials and seek local perspectives on the debates? Examples might include debates about use of standard of care versus placebo for comparator arms, emergent evidence that might change the trial landscape, and issues of equity related to potential ancillary care needs of trial participants. Please check all that apply.<br>• If (3) then value=0<br>• Else value=sum of checked items	• Global debates have been tracked and discussed by staff at our site in the last 12 months<br>• Local perspectives on debates have been sought by staff at our site in the last 12 months<br>(3) Our site has not actively tracked such debates in the last 12 months
	Intensity score for Mapping<br>• Rationale: greater use of a variety of mapping practices indicates greater understanding of context	• Value=sum of values for Mapping practices (Q3.2-Q3.9)	

**TOC Framework component**	**Indicators**	**Sub-measures within indicators**<br>*Note: includes questions as worded in survey & logic for calculating value based on responses*	**Response items within measures**
	Range: 0-38		
Shared learning	[Non-indicator item]	Q4.1 The next set of questions focus on shared learning. Shared Learning is a strategy that research sites can use to increase shared knowledge between researchers and community stakeholders, including increased literacy on community context, the research context, and TB.	
	Learn from local stakeholders<br>• Rationale: direct participation of local stakeholders in research team meetings indicates shared learning<br>• Range: 0-5	Q4.2 In the last 12 months have local community representatives and stakeholders participated in research team meetings at your site in any of the following ways? Please check all that apply.<br>• Value=sum of checked items	• Presented information to the research team<br>• Provided training for the research team<br>• Engaged in discussion on research team agenda items<br>• Reported back to community-based constituencies about issues discussed by the research team<br>• Reported feedback from community-based constituencies to the research team about specific issues
	Learn about global topics<br>• Rationale: Shared learning about global context among research staff is greater when staff are actively supported in accessing learning opportunities<br>• Range: 0-4	Q4.3 Thinking about all of the TB trials research staff at your site, how many in the last 12 months were provided time and resources to improve their understanding of global topics related to TB research? This could include participation in open-forum email lists, websites, webinars, and participation in TB-related conferences.<br>• If (1) then value=0<br>• If (2) then value=1<br>• If (3) then value=2<br>• If (4) then value=3<br>• If (5) then value=4	(1) None<br>(2) One or two<br>(3) Some but fewer than half<br>(4) More than half but not all<br>(5) All
	Learn from non-local dissemination opportunities<br>• Rationale: Shared learning by researchers and stakeholders is greater when structured opportunities exist to share information	Q4.4 In the last 12 months has your site held at least one structured meeting where research team and local stakeholders shared information from a meeting, conference or workshop held at the national, regional or global level?<br>• If (1) then value=1<br>• If (2) then value=0	(1) Yes<br>(2) No

**TOC Framework component**	**Indicators**	**Sub-measures within indicators** *Note: includes questions as worded in survey & logic for calculating value based on responses*	**Response items within measures**
	disseminated at non-local events<br>• Range: 0-1		
	Learn engagement<br>• Rationale: Training in engagement increases research team capacity and skills needed for shared learning<br>• Range: 0-4	Q4.5 In the last 12 months how many staff at your site have undergone any formal training related to community and stakeholder engagement in research?<br>• If (1) then value=0<br>• If (2) then value=1<br>• If (3) then value=2<br>• If (4) then value=3<br>• If (5) then value=4	(1) None<br>(2) One or two<br>(3) Some but fewer than half<br>(4) More than half but not all<br>(5) All
	Learn needs<br>• Rationale: Use of a needs assessment will help identify opportunities to improve shared learning<br>• Range: 0-1	Q4.6 In the last 12 months has your site undertaken a needs assessment of community stakeholder and research staff to determine potential capacity-building needs with regard to levels of knowledge and expertise needed for effective engagement with each other?<br>• If (1) then value=1<br>• If (2) then value=0	(1) Yes<br>(2) No

**TOC Framework component**	**Indicators**	**Sub-measures within indicators**<br>*Note: includes questions as worded in survey & logic for calculating value based on responses*	**Response items within measures**
	Learn about local context<br>• Rationale: The more staff who have knowledge of local context, the greater their community literacy<br>• Range: 0-16<br><br>Learn about trial context<br>• Rationale: The more staff who have knowledge about TB clinical trials, the greater their capacity to increase community literacy about TB research<br>• Range: 0-20	Q7.2 Thinking about all the TB trials research staff at your site, how many could speak knowledgeably about the following topics?<br>• For staff knowledge response, set values as follows:<br>○ If (1) then value=4<br>○ If (2) then value=3<br>○ If (3) then value=2<br>○ If (4) then value=1<br>○ If (5) then value=0<br>○ If (6) then value=0<br><br>• For local context<br>○ Value=sum of staff knowledge response values for each topic<br>• For trial context<br>○ Value=sum of staff knowledge response values for each topic	Local context topics:<br>• Cultural norms, practices and beliefs of your site's research participants<br>• Social, political and economic circumstances of your site's research participants<br>• The diversity of viewpoints held by TB research stakeholders in the local community<br>• The diversity of viewpoints held by TB research stakeholders in your country<br><br>Trial context topics:<br>• The scientific research process that guides TB clinical trials<br>• The kinds of considerations included in the design of a TB clinical trial<br>• How to collect TB trial data<br>• How to analyze TB trial data<br>• How to explain the findings from a TB clinical trial<br><br>Staff knowledge response scale:<br>(1) All<br>(2) Most<br>(3) Some<br>(4) Few<br>(5) None<br>(6) Don't know
	Intensity score for Shared Learning<br>• Rationale: greater use of a variety of learning practices indicates greater shared knowledge between researchers and community stakeholders<br>Range: 0-51	Value=sum of values for Shared Learning practices (Q4.2-Q4.6 and Q7.2)	

**TOC Framework component**	**Indicators**	**Sub-measures within indicators**<br>*Note: includes questions as worded in survey & logic for calculating value based on responses*	**Response items within measures**
Responsible advocacy	[Non-indicator item]	Q5.1 The next set of questions focus on responsible advocacy. Responsible advocacy refers to actions that research sites might take to increase awareness, political will, and resources for TB clinical trials and community engagement.	
	Advocacy for TB trials<br>• Rationale: Activities increase knowledge and awareness of why TB research is important<br>• Range: 0-5	Q5.2 In the last 12 months has your site taken any of the following steps in support of TB trials research? Please check all that apply.<br>• Value=sum of checked items	• Identified one or more stakeholders who are effective and trusted champions of TB research<br>• Identified one or more stakeholders as "translational advocates" who can go back and forth between constituents to take the pulse of different stakeholders<br>• Developed a media campaign to generate support for TB research (not specifically for recruiting participants into a clinical trial)<br>• Supported a grassroots campaign by community members or other stakeholders in their efforts to develop a media campaign to generate support for TB research<br>• Provided educational briefings to policy makers on issues relevant to TB trials research
Deliberation	[Non-indicator item]	Q6.1 The next set of questions focus on deliberation. Deliberation is a strategy that includes formal discussion and negotiation with all stakeholders to ensure options for mutual gain are pursued when trade-offs in highly valued but conflicting principles may be needed.	
	Deliberation regarding a conflict<br>• Rationale: Indicator that a conflict or tension occurred<br>• Range: 0-1	Q6.2 In the last 12 months has your site had a situation where there was a conflict or tension between research principles and/or principles of importance to other stakeholders in the local context?<br>• If (1) then continue and value=1<br>• If (2) skip to Deliberation regarding a hypothetical conflict and value=0	(1) Yes<br>(2) No
		*If **Yes** to conflict question (Q6.2) then answer next question. Else skip to (Q6.5).*	

**TOC Framework component**	**Indicators**	**Sub-measures within indicators**<br>*Note: includes questions as worded in survey & logic for calculating value based on responses*	**Response items within measures**
	Deliberation structure<br>• Rationale: Use of structured opportunity indicates stronger partnering with stakeholders<br>• Range: 0-1	Q6.3 When this conflict or tension arose did you seek or provide a structured opportunity for all concerned stakeholders to communicate their concerns and perspectives?<br><br>Deliberation structure:<br>• If (1) or (2) then value=1<br>Else value=0<br><br>*NOTE: See Outcomes section below for calculation of Conflict outcome measure for this question.*	(1) Yes, and the opportunity led to a successful resolution<br>(2) Yes, but we were not able to reach agreement on a resolution<br>(3) No, but we were able to resolve the issue through other means<br>(4) No, and we have ongoing problems or concerns about it<br>(5) No, and the problem seems to have faded away on its own
	Deliberation strategy<br>• Rationale: The more mechanisms used, then the greater support there was for decision-making to be equitably shared by researchers and concerned stakeholders<br>• Range: 0-6	Q6.4 During the structured opportunity where all concerned stakeholders met, were any of the following strategies used? Please check all that apply.<br>• If (1) or (8) then value=0; else<br>• Sum of following values if item checked:<br>○ If (2) then value=1<br>○ If (3) then value=1<br>○ If (4) then value=1<br>○ If (5) then value=1<br>• Add following values to sum if item is *not* checked:<br>○ If not (6) then value=1<br>○ If not (7) then value=1<br><br>*NOTE: Items (6) & (7) are reverse scored because use of these strategies indicates the decision making related to the conflict was out of the hands of concerned stakeholders. Therefore, non-use of these strategies is reflective of greater use of Deliberation with stakeholders.*	(1) A structured opportunity was not used<br>(2) Explicit norms or rules for discussion were established, e.g., stay on topic, not interrupt, give everyone a chance to speak, turn-taking<br>(3) A neutral facilitator was used throughout the process<br>(4) A process was used where authority was shared equally by all stakeholders<br>(5) A decision-making mechanism was used where all stakeholders participated in the decision (e.g., a vote was taken)<br>(6) A neutral party arbitrated the decision after hearing arguments from all sides (e.g., an ethics committee)<br>(7) Recommendations were presented to your site's leadership, the sponsor or the funder for a final decision<br>(8) None of the above

TOC Framework component	Indicators	Sub-measures within indicators<br>*Note: includes questions as worded in survey & logic for calculating value based on responses*	Response items within measures
	Deliberation as a future strategy<br>• Rationale: The more explicitly the strategy includes plans for input beyond the research team, then the greater support there is likely to be for decision-making to be equitably shared by researchers and concerned stakeholders<br>• Range: 0-5	Q6.5 Imagine that a conflict or tension were to arise tomorrow between research principles and/or principles of importance to other stakeholders in the local context. Which of the following statements best describes how your site would first respond?<br>• If (1) then value=1<br>• If (2) then value=0<br>• If (3) then value=3<br>• If (4) then value=5<br>• If (5) then value=4<br>• If (6) then value=2	(1) Our research site has written procedures in place for this kind of event and we would consult them<br>(2) The principle investigator for the research in question would determine the appropriate steps to take<br>(3) We would consider which stakeholders were involved and then decide how to respond<br>(4) We would conduct a rapid assessment to map out the issues and who would be affected by a final decision<br>(5) We have a CAB-type mechanism that handles these kinds of things<br>(6) Our research site has not dealt with something like this before so we would seek expert advice
**Outcomes**			
	Site challenges<br>• Rationale/hypothesis: Use of the powerful strategies will help research sites to align their work better with the local context and result in fewer challenges for research sites<br><br>Range:<br>• Mutual gain -4 to +2<br>• Transparency and integrity -2 to +1<br>• Shared knowledge -2 to +1	Q7.3 Please indicate the extent to which each of the following has been a challenge for your research site in the last 12 months.<br><br>Response scale value for each item:<br>• If (Not a challenge) then value= 1<br>• If (Somewhat of a challenge) then value= -1<br>• If (A major challenge) then value= -2<br><br>Mutual gain<br>• Sum value for Items (1) & (2)<br><br>Transparency and integrity<br>• Value for Item (3)<br><br>Shared knowledge<br>• Value for Item (4)	(1) Competition with the public health system for human resources (i.e., qualified staff)<br>(2) Infrastructure built for TB trials uses standards relevant for the local health system<br>(3) Establishing effective communication networks for reporting and monitoring of TB cases identified<br>(4) Ensuring local stakeholder understanding of TB disease, treatment and prevention<br><br>Response scale:<br>• Not a challenge<br>• Somewhat of a challenge<br>• A major challenge

**TOC Framework component**	**Indicators**	**Sub-measures within indicators**<br>*Note: includes questions as worded in survey & logic for calculating value based on responses*	**Response items within measures**
	Conflict outcome<br>• Rationale/Hypothesis: Use of the powerful strategies will lead to better ability to resolve a conflict or tension<br>Range: 0-2	Q6.3 When this conflict or tension arose did you seek or provide a structured opportunity for all concerned stakeholders to communicate their concerns and perspectives?<br><br>Conflict outcome:<br>• If (1) then value=2<br>• If (3) then value=1<br>• Else value=0	(1) Yes, and the opportunity led to a successful resolution<br>(2) Yes, but we were not able to reach agreement on a resolution<br>(3) No, but we were able to resolve the issue through other means<br>(4) No, and we have ongoing problems or concerns about it<br>(5) No, and the problem seems to have faded away on its own
	Trials implemented<br>• Rationale/Hypothesis: Use of the powerful strategies will generate the awareness, political will, and/or resources needed to implement clinical trials.<br>• Range: open	Q7.4 Has your site EVER implemented a TB clinical trial? By implement we mean your site was activated and enrolled participants.<br>• If (1) then go to next question<br>• If (2) then value=0<br><br>Q7.5 How many TB clinical trials have your site implemented? Include current ongoing trials in the total.<br>• Value=number reported	(1) Yes<br>(2) No

	Trial outcomes<br>• Rationale/Hypothesis: Use of the powerful strategies will, over the long term, lead to more positive outcomes reflective of the GPP-TB goal (access, social value, acceptability). Conversely, negative outcomes indicate potential long-term harm associated with use of the powerful strategies.<br><br>• Range:<br>○ Effective products available 0 to 1<br>○ Access -5 to 6<br>○ Social value -1 to 4<br>○ Acceptability -3 to 2	Q7.6 Thinking about the most recent clinical trial that your site implemented, which of the following are true for that trial? Please check all that apply.<br><br>Intermediate & long-term outcome:<br>• Effective products available<br>○ If any of (9) through (12) then value= 1, else 0<br><br>Ethical Goal outcomes:<br>• If (1) then<br>○ Access= -1<br>• If (2) then<br>○ Access= -1<br>• If (3) then<br>○ Access= -1<br>• If (4) then<br>○ Access= -1<br>○ Social value= -1<br>• If (5) then<br>○ Access= +1<br>○ Social value= +1<br>• If (6) then<br>○ Social value= +1<br>• If (7) then<br>○ Social value= +1<br>• If (8) then<br>○ Access= -1<br>○ Acceptability= -1<br>• If (9) then<br>○ Access= +1<br>• If (10) then<br>○ Access= +1<br>○ Acceptability= -1<br>• If (11) then<br>○ Access= +1<br>○ Acceptability= -1<br>• If (12) then<br>○ Access= +1<br>○ Acceptability= +1<br>• If (13) then<br>○ Access= +1<br>○ Social value= +1	(1) Our site was not able to recruit the target number of participants<br><br>(2) Our site was not able to retain and follow up with the target number of participants<br><br>(3) Our site was closed early due to recruitment or retention problems but the trial continued at other sites<br><br>(4) The trial was closed early<br><br>(5) The trial was successfully completed<br><br>(6) The trial demonstrated efficacy in one or more treatment arms<br><br>(7) The trial ultimately led to new TB treatment or prevention guidelines<br><br>(8) The experimental drug tested in the trial is not suitable for use in the local context<br><br>(9) The experimental drug tested in the trial is now available and affordable in our local community<br><br>(10) The experimental drug tested in the trial is available but many providers refuse to use it<br><br>(11) The experimental drug tested in the trial is available but many patients refuse or fail to use it as directed<br><br>(12) The experimental drug tested in the trial is available and successfully used by providers and patients<br><br>(13) The trial results have contributed to lower TB morbidity and mortality in our country

TOC Framework component	Indicators	Sub-measures within indicators<br>*Note: includes questions as worded in survey & logic for calculating value based on responses*	Response items within measures
		○ Acceptability= +1<br><br>Sum response values above for<br>• Access<br>• Social value<br>• Acceptability	
